# Noble Metal Nanoparticles Stabilized by Hyper-Cross-Linked Polystyrene as Effective Catalysts in Hydrogenation of Arenes

**DOI:** 10.3390/molecules26154687

**Published:** 2021-08-03

**Authors:** Elena S. Bakhvalova, Arina O. Pinyukova, Alexey V. Mikheev, Galina N. Demidenko, Mikhail G. Sulman, Alexey V. Bykov, Linda Z. Nikoshvili, Lioubov Kiwi-Minsker

**Affiliations:** 1 Regional Technological Centre, Tver State University, Zhelyabova Str., 33, 170100 Tver, Russia; bakhvalova.es@mail.ru; 2 Department of Biotechnology, Chemistry and Standardization, Tver State Technical University, A.Nikitina Str., 22, 170026 Tver, Russia; yourchemist@mail.ru (A.O.P.); exmahina.leha@gmail.com (A.V.M.); xt345@mail.ru (G.N.D.); sulmanmikhail@yandex.ru (M.G.S.); bykovav@yandex.ru (A.V.B.); 3 Department of Basic Sciences, Ecole Polytechnique Fédérale de Lausanne (GGRC-ISIC-EPFL), CH-1015 Lausanne, Switzerland

**Keywords:** polymeric catalysts, hydrogenation of arenes, noble metals, hyper-cross-linked polystyrene, catalyst stability

## Abstract

This work is addressing the arenes’ hydrogenation—the processes of high importance for petrochemical, chemical and pharmaceutical industries. Noble metal (Pd, Pt, Ru) nanoparticles (NPs) stabilized in hyper-cross-linked polystyrene (HPS) were shown to be active and selective catalysts in hydrogenation of a wide range of arenes (monocyclic, condensed, substituted, etc.) in a batch mode. HPS effectively stabilized metal NPs during hydrogenation in different medium (water, organic solvents) and allowed multiple catalyst reuses.

## 1. Introduction

Hydrogenation of different arenes, mono- and polycyclic, and their mixtures are highly used in modern industries: refining of fuels; hydrogenation of benzene (BZ) to cyclohexane (CH) for the synthesis of caprolactam and toluene (TOL) to methylcyclohexane (MCH); hydrogenation of naphthalene (NL) in the synthesis of tetralin (TL) and decalin (industrial hydrogen donors and solvents used for extraction); hydrogenation of aniline (AN) and its derivatives for the production of anticorrosive additives. All these reactions are energy-intensive, large-capacity petrochemical processes that produce reagents for chemical and pharmaceutical industries, and are used for reduction of residuals in fuels and lubricants [1,2,3,4].

Most methods for the production of functionalized and nonfunctionalized cycloalkanes from BZ, NL, polyarenes, and their derivatives are based on gas-phase catalytic processes. Such processes have a number of disadvantages: high temperatures of vapor–gas mixture and nonuniform heat removal resulting in catalyst deactivation; catalyst abrasion and metal loss in the case of fluidized bed reactors. Liquid-phase hydrogenation of arenes is carried out at much lower temperatures using catalytic systems based mainly on nickel or noble metals (Pt, Pd, Ru, Rh) deposited on inorganic supports, but is less studied [5,6,7].

The conditions for hydrogenation of aromatics are selected depending on the concentration of sulfur-containing impurities in the raw material [8]. If BZ is almost free of sulfur compounds, the reaction is carried out in the presence of low-temperature catalysts at low hydrogen pressure. The BZ containing sulfur impurities must be hydrogenated over sulfur-resistant catalysts under more harsh conditions. Nickel catalysts, in comparison with platinum group metals, are preferred for hydrogenation of sulfur-containing raw materials since they are more resistant to sulfur poisoning.

The use of different inorganic supports (e.g., alumina, silica, zeolites) allows high catalyst activity and selectivity; however, their acidic properties provoke carbon deposition. Moreover, active metal phase deposited on inorganic supports is often unstable and undergoes sintering, resulting in catalyst deactivation. The use of polymeric supports brings a big advantage: the catalytically active phase is well stabilized within cavities distributed over the entire volume of the polymer and becomes fully accessible to reactants when the support swells. Moreover, polymeric supports are usually hydrophobic, and are suitable for organic solvents.

In this work, for the first time, hydrogenation by molecular hydrogen of a wide range of arenes (monocyclic and condensed, substituted and nonsubstituted) was studied over noble metal (Pd, Pt, Ru) NPs supported on hyper-cross-linked polystyrene (HPS) of two types: MN270 (nonfunctionalized) and MN100 (bearing tertiary amino groups and designated as HPS-NR_2_).

It is noteworthy that, to date, there are only a few reports on the use of polymer-based catalysts in hydrogenation of arenes. The most recent work, of Davankov et al. [9], was devoted to the hydrogenation of BZ, TOL, TL, and phenol (PL) using Pd NPs supported on the HPS of MN200-type (analogue of MN270, but with larger meso-/macro-pores with a mean diameter of 70 nm). It was shown that, using supercritical CO_2_ or methylene chloride as a solvent, Pd/MN200 successfully catalyzed hydrogenation of aromatics in the temperature range of 55–150 °C. Moreover, Pd/HPS catalysts exhibited higher activity compared to traditional catalysts containing an equivalent amount of Pd [10].

## 2. Results

**Figure 1:**
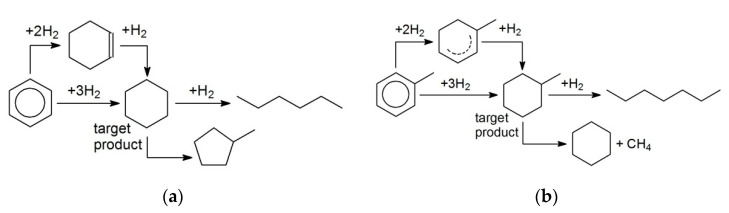
Simplified schemes of selective hydrogenation of BZ to CH (**a**) and TOL to MCH (**b**).

First, the hydrogenation of BZ to cyclohexane (CH) was studied (the simplified reaction scheme is shown in Figure 1a) at variation of solvent nature, BZ concentration, and catalyst composition (polymer type, metal loading, and nature). It is noteworthy that different side products can be found during BZ hydrogenation [5,7], but their formation is associated with the acid–base properties of the supports used. In the case of polymeric supports (in particular, HPS), 100% selectivity with respect to CH was observed irrespective of the metal nature and reaction conditions (see Table 1).

**Figure 2:**
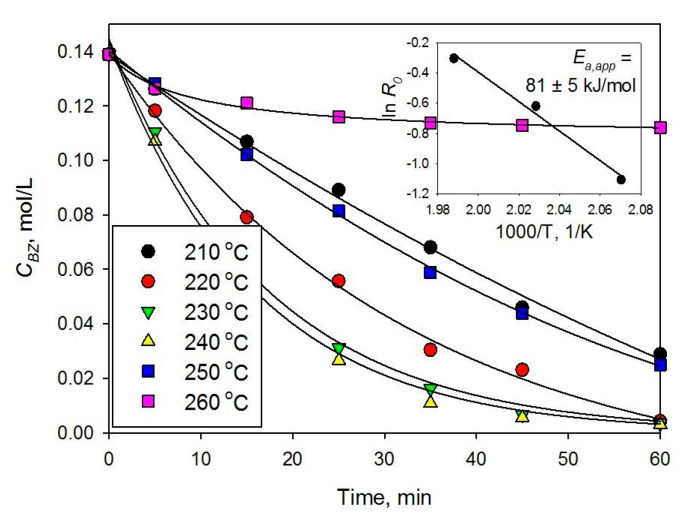
Effect of temperature on hydrogenation of BZ to CH over 1% Pt/HPS (5 MPa, catalyst loading 100 mg); inset shows the Arrhenius plot for initial reaction rate, *R*_0_.

Most of BZ hydrogenations were carried out in hexane medium at 230 °C, which was found as optimal temperature during the reaction study with 1% Pt/HPS (see Figure 2 and Table 1, #1). As can be seen in Figure 2, the initial rate of BZ transformation gradually increases with the temperature increase from 210 °C up to 230 °C. At 240 °C, the rate of BZ hydrogenation attains the maximum, and the following increase of temperature up to 260 °C results in a sharp decrease of the reaction rate (note that the critical point of hexane is 234.5 °C at 3.02 MPa). Recently, we have found that hydrogenation of NL to TL in hexane can effectively proceed at 250 °C and at 6 MPa of overall pressure [11]. 

The strong decrease of hydrogenation rate while shifting to supercritical hexane, in the case of BZ in contrast to NL, can be due to the difference in adsorption of mono- and dicyclic arenes. In general, if the metal NPs are large enough, condensed arenes should adsorb stronger. Nevertheless, in the range of 210–230 °C, the apparent activation energy of BZ hydrogenation was calculated as *E_a,app_* = 81 ± 5 kJ/mol (Figure 2), which is in agreement with the data reported elsewhere [12,13]. It is noteworthy that for calculation of *E_a,app_*, the initial reaction rate *R*_0_ was obtained as a tangent of the slope on the kinetic curve related to the metal concentration (see Section 4.3). We can suppose that the coverage of metal surface by hydrogen is low, which is typical for small conversions where concentration of substrate is still high [14]. Thus, with high selectivity to target product and without any influence of diffusion, the irreversible addition of hydrogen atoms to the adsorbed substrate can be considered as a rate-determining step.

For the catalyst 1% Pt/HPS, the effect of reuse was also studied (Table 1, #2,3). It was found that nearly double a decrease of the initial reaction rate (*R*_0_) takes place at the second reaction run. The polymeric matrix of HPS remained stable during the experiments. Low-temperature nitrogen physisorption revealed that the specific surface area (SSA) determined according to the BET model was nearly the same for both the initial (934 m^2^/g) and the used (955 m^2^/g) catalyst. Using the XPS method, it was found that there is a slight change in the ratio between different forms of Pt on the catalyst surface: the increase of the content Pt^0^ (binding energy (BE) of Pt 4f_7/2_ is 71.5 eV) was observed (from 77 at.% up to 87 at.%) with concomitant decrease of the content of PtO (BE of Pt 4f_7/2_ is 72.9 eV) from 23 at.% to 13 at.%; however, these changes cannot explain the significant decrease in catalytic activity for the second use. The reason can be due to the formation of carbonaceous deposits on the surface of Pt NPs, which often causes the catalyst deactivation during the hydrogenation of arenes [13,15]. However, Pt content (determined by the XPS) on the catalyst surface even increases after the catalytic experiment from about 0.3 at.% up to 0.5 at.%. Thus, we can suppose that the observed drop in catalytic activity is likely due to the changes in morphology of the catalytically active phase, i.e., agglomeration of NPs. It is known that the sizes of Pt NPs have a crucial effect on the kinetics of BZ hydrogenation [16].

**Figure 3:**
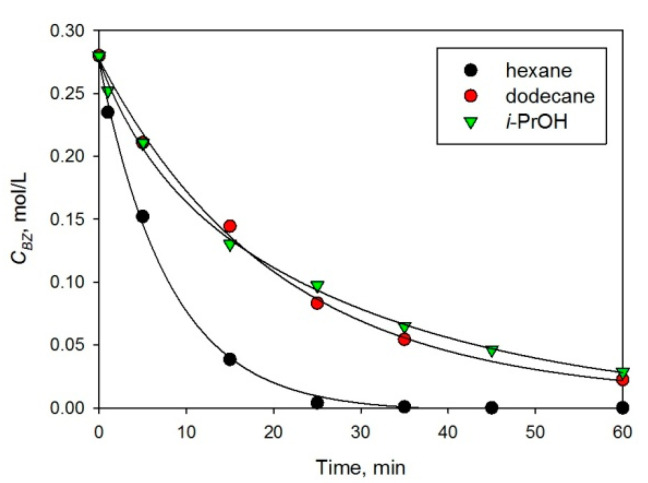
Solvent effect on BZ hydrogenation to CH over 2% Pt/HPS-NR_2_ (230 °C, 5 MPa, *C_BZ_*_,0_ = 0.28 mol/L, catalyst loading 100 mg).

The effect of solvent nature was studied using 2% Pt/HPS-NR_2_ as catalyst (see Figure 3 and Table 1, #5–7). As can be seen from Figure 3, the use of hexane as a solvent provides the highest rate of BZ transformation, although at the chosen temperature (230 °C), the vapor pressure of hexane is rather high—about 2.7 MPa. In the case of *i*-PrOH and dodecane, the reaction rate was noticeably lower, compared to hexane. It is noteworthy that at 230 °C, *i*-PrOH has vapor pressure higher than 4 MPa, which drastically decreases hydrogen partial pressure (overall pressure in the reactor was 5 MPa). Nevertheless, the rate of BZ hydrogenation in *i*-PrOH medium was comparable with those in dodecane as a solvent, possibly due to the ability of *i*-PrOH to serve as an effective hydrogen donor [17].

At the chosen temperature (230 °C) in hexane medium, the effect of metal nature was studied (Table 1, #7–9). It was found that Pt is the most effective metal as it provides a 3–6-fold higher initial reaction rate compared to Pd and Ru (note that RuO_2_ is an active phase of 2% Ru/HPS-NR_2_).

In contrast to BZ, TOL was three-fold faster hydrogenated exclusively with MCH (Figure 1b) (Table 1, #4 vs. 12) using 1% Pt/HPS-NR_2_ in dodecane. This result is unusual, since, according to other reports, the rates of BZ and TOL hydrogenation are often comparable [18,19].

**Figure 4:**
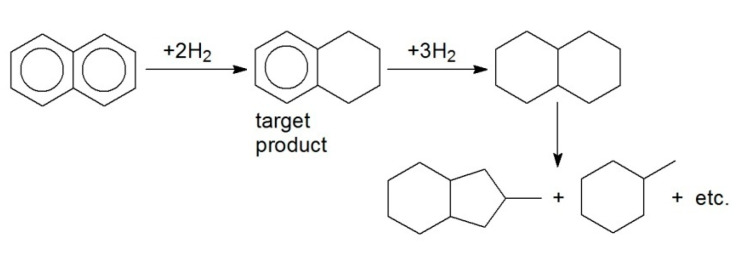
Simplified scheme of selective hydrogenation of NL to TL.

**Figure 5:**
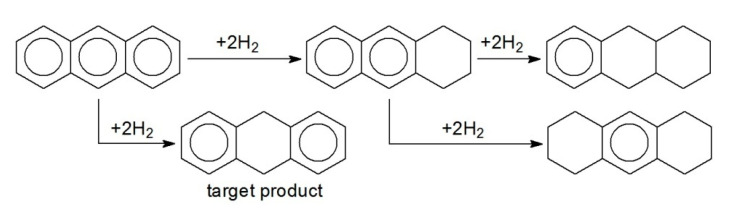
Simplified scheme of selective hydrogenation of ANC to 9,10-DANC.

Hydrogenation of naphthalene (NL) and anthracene (ANC) was carried out over 1% Pt/HPS-NR_2_. NL was found to be selectively hydrogenated to target TL (Figure 4), while in the case of ANC, 9,10-dihydroanthracene (9,10-DANC) was the main reaction product (Figure 5). Pt-containing catalysts were shown to be stable in four consecutive runs of NL and ANC hydrogenation without any noticeable loss of activity and selectivity under the chosen reaction conditions.

It was also found that the initial hydrogenation rate (*R*_0_) increases in the order: NL < BZ < ANC (Table 1, #4,10,11). In contrast to the data reported by Rautanen et al. [20], who claimed that in condensed aromatics the first ring of diaromatic compounds (i.e., NL) is more reactive than the second one, in the case of Pt/HPS catalysts, NL was less reactive compared to BZ.

At the same time, in the ANC hydrogenation, the main side product was 1,2,3,4-tetrahydro-ANC (about 9% was accumulated during 60 min), and no compounds with higher degree of saturation were found. This result indicates that the rate of side-ring hydrogenation is low and can be due to the peculiarities of arenes adsorption on Pt NPs. Higher reactivity of ANC in comparison with BZ is likely due to the fact that C_9_ and C_10_ atoms of the central ring in ANC have partially higher electron density due to the influence of two side rings, making these carbon atoms more active for hydrogenation.

**Figure 6:**
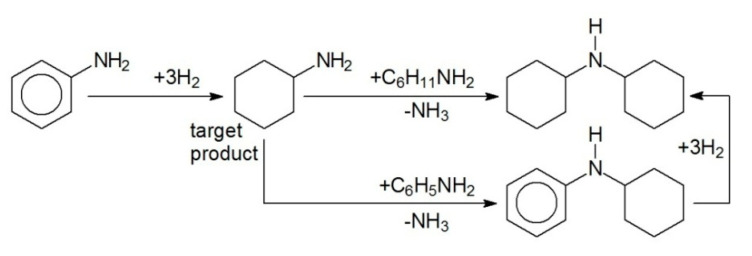
Simplified scheme of selective hydrogenation of AN to CHA.

**Figure 7:**
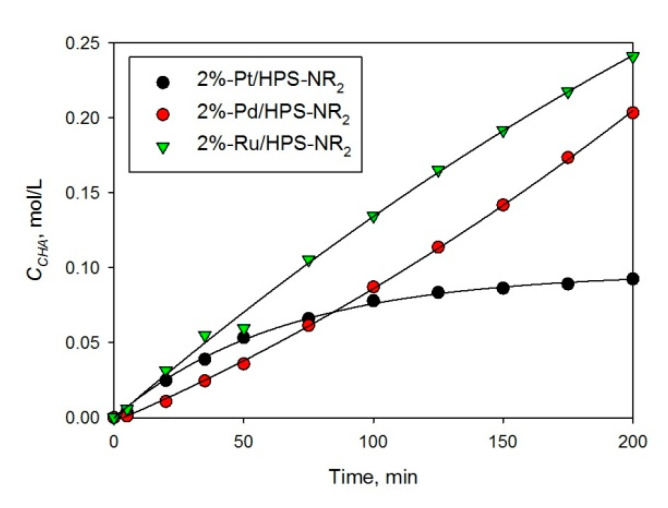
CHA accumulation using different noble metal NPs supported on HPS-NR_2_ (150 °C, 3 MPa, *C_AN_*_,0_ = 0.41 mol/L, catalyst loading 100 mg).

Hydrogenation of different substituted arenes was also studied, taking AN, PL, phenylacethylene (PHA), and propiophenone (PP) as models. In the selective hydrogenation of AN to cyclohexylamine (CHA) (see Figure 6), HPS-supported Pt NPs were the most effective noble metal-containing catalysts independent of the polymer type and metal loading (Figure 7 and Table 2, #1–5). In spite of the fact that numerous products can be formed during the AN hydrogenation [5], we observed only two side products depicted in Figure 6.

**Figure 8:**
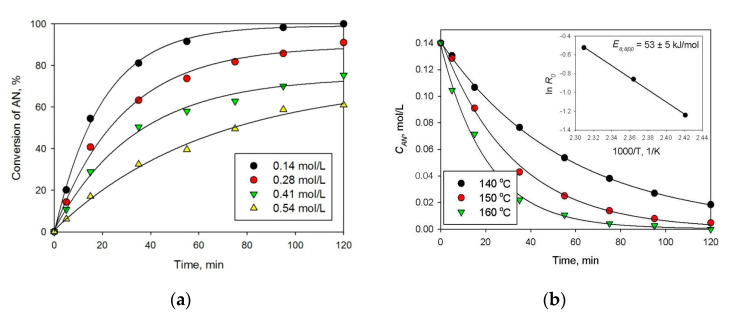
Effect of AN initial concentration (**a**) and temperature (**b**) on selective hydrogenation of AN to CHA using 1 wt.% Pt/HPS (2 MPa, catalyst loading 100 mg).

However, in spite of the high initial reaction rate, Pt-containing HPS catalysts deactivated, especially at high initial concentrations of AN, which is seen from the shape of kinetic curves (see Figure 8a). At the same time, the apparent activation energy was 53 ± 5 kJ/mol (Figure 8b), which is comparable with literature data [21]. Moreover, it was found that 1% Pt/HPS is rather stable at the reuse (Table 2, #1,2); although the initial reaction rate slightly decreases, conversion and selectivity achieved in 120 min of the reaction remains the same. Observed loss of activity during the same reaction run can be due to strong adsorption of AN and products on the metal surface, acting similar to poisons [22,23], but this effect seems to take place only in situ under the reaction conditions.

**Figure 9:**
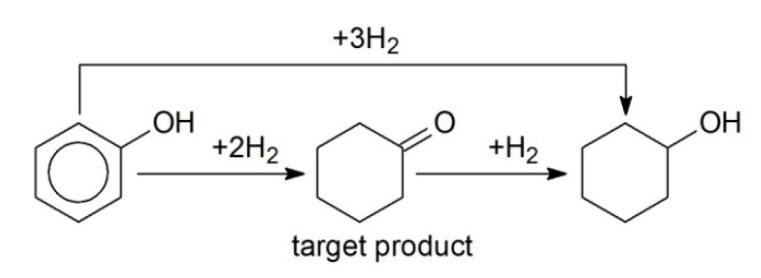
Scheme of selective hydrogenation of PL to CHN.

Hydrogenation of PL, PHA, and PP traditionally is carried out using Pd-containing catalysts. In the case of PL hydrogenation (Figure 9), different reaction conditions (solvents, temperatures, and pressures) can be used [24]. Earlier, we showed that PL can be successfully hydrogenated in a gas phase in a fixed-bed reactor over 0.5% Pd/HPS catalyst [25]. In this work, an autoclave-type reactor was used, and three solvents (Table 2, #6–8) at 150 °C and hydrogen partial pressure of 2 MPa (comparable with some other works) [26] were tested. As can be seen from Table 2 (#6–8), water is the best solvent, allowing the highest hydrogenation rate, while the selectivity over 2% Pd/HPS-NR_2_ with respect to cyclohexanone (CHN) is moderate (91% to CHL at 50% of PL conversion) and needs further improvement.

**Figure 10:**
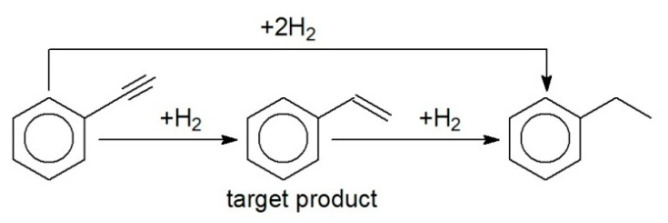
Scheme of selective hydrogenation of PHA to STY.

**Figure 11:**
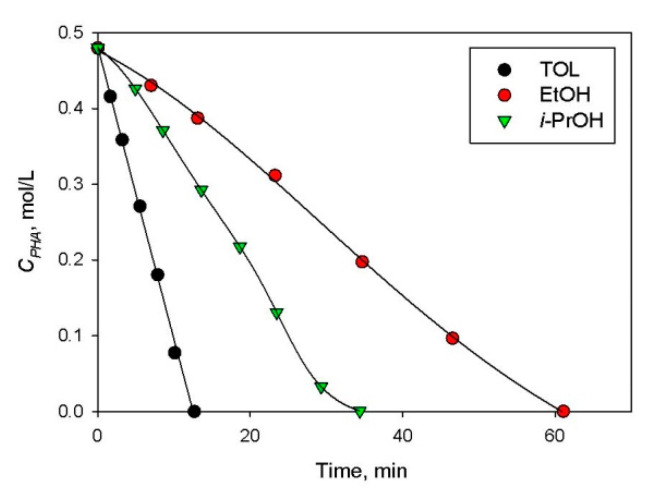
Solvent effect on PHA hydrogenation to STY over 1 wt.% Pd/HPS-NR_2_ (ambient H_2_ pressure, *C_PHA_*_,0_ = 0.48 mol/L, catalyst loading 30 mg).

PHA can be easily hydrogenated to styrene (STY) (Figure 10) under mild reaction conditions (low temperatures and ambient hydrogen pressure). Nevertheless, it is very difficult to provide high selectivity with respect to STY [27] due to exceptionally high reactivity of triple and double carbon–carbon bonds, when one of the hydrogen atoms is substituted in a benzene ring. In the case of 1 wt.% Pd/HPS-NR_2,_ TOL was found to be the best solvent, providing exceptionally high activity (Figure 11) and 88% selectivity with respect to STY at close to 100% PHA conversion (Table 2, #9–11), which is comparable with other reports [28]. It is noteworthy that different temperatures were tested which were about 10–15 °C lower than the boiling point of the corresponding solvent. The higher the temperature, the higher the selectivity to olefinic product; however, hydrogenation rate usually goes through a maximum, which is 90 °C for TOL [29].

**Figure 12:**
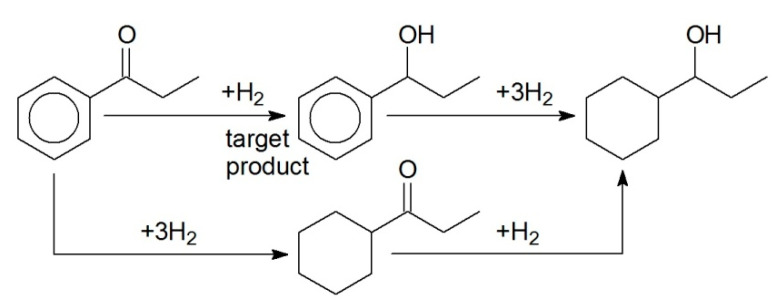
Scheme of selective hydrogenation of PP to PPL.

PP was also hydrogenated using Pd-containing catalyst (1 wt.% Pd/HPS-NR_2_). Phenylpropanol (PPL) was found to be the main reaction product (Figure 12).

**Figure 13:**
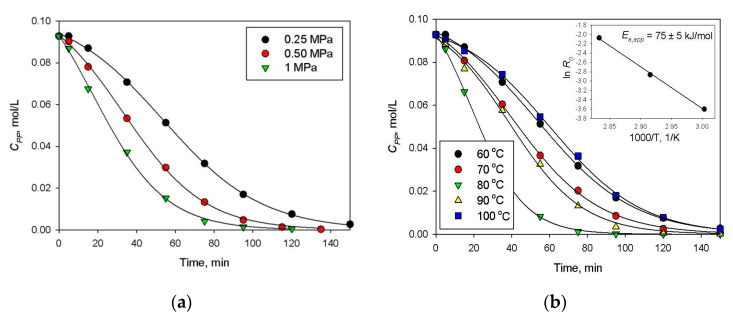
Effect of overall pressure (**a**) and temperature (**b**) on selective hydrogenation of PP to PPL using 1 wt.% Pd/HPS-NR_2_ (*C_PP_*_,0_ = 0.09 mol/L, catalyst loading 100 mg).

Variation of overall pressure (Figure 13a) allowed determining the formal kinetic parameter with respect to hydrogen, which was close to the unit. The study of the temperature effect was carried out at 0.25 MPa (Figure 13b). It was found that the reaction rate increased with the increase of temperature from 60 °C up to 80 °C (in this range, *E_a,app_* = 75 ± 5 kJ/mol, calculated taking into account the same assumptions as in the case of BZ hydrogenation). Further increase of temperature resulted in noticeable decrease of the PP hydrogenation rate due to the high vapor pressure of the solvent (hexane). Thus, 80 °C can be considered as the optimal temperature (see also Table 2, #12), which provided 98% conversion of PP (for 240 min) and exclusive formation of PPL (100% selectivity).

It is noteworthy that, across the whole range of chosen temperatures, 98% selectivity with respect to PPL was found at 0.25 MPa overall pressure, which is a very promising result in comparison with other reported data [30,31]. A slight decrease of selectivity (at 99% of PP conversion) was observed at higher pressures: 97% at 5 MPa, and about 95% at 10 MPa.

## 3. Discussion

The results of our work reported here demonstrate that noble metal NPs are effectively stabilized within polymeric networks of HPSs. HPSs of different types and properties (SSA, hydrophobicity, functional groups, etc.) are commercially available at a relatively low price while being chemically and mechanically stable. Moreover, contrary to most inorganic solids (such as alumina, silica, zeolites, etc.), HPSs have excellent wettability in organic solvents and do not present surface acidity, which is known to be responsible for the formation of a number of byproducts and carbon deposits, leading to catalyst deactivation.

HPS-based catalysts containing NPs of different metals (Pt, Pd, Ru) were prepared via simple wet-impregnation and thoroughly characterized by different physical and chemical methods. Pd NPs had mean diameter of 2.6 ± 0.4 nm and were evenly distributed through the polymeric network irrespective of the polymer type, palladium loading, or reduction method. The 2% Pd/HPS-NR_2_ with hexane as solvent demonstrated extraordinary catalytic properties in BZ to CH hydrogenation, showing 100% CH selectivity at close to 100% conversion of BZ. Pd NPs stabilized by HPS also showed excellent results in PP hydrogenation, giving almost exclusively the target PPL. Pt NPs had slightly bigger mean diameter (3.9 ± 1.7 nm) compared to Pd and also some aggregates with diameters of >11 nm. The 2% Pt/HPS-NR_2_ also demonstrated 100% CH selectivity at up to full BZ conversion, but outperformed the Pd-based catalyst in terms of a twofold higher reaction rate under the same reaction conditions. The 2% Ru/HPS-NR_2_ contained small NPs of RuO_2_ of about 2–4 nm in diameter, which were distributed nonuniformly, closer to the outer surface of the polymer granule, and showed much lower performance in hydrogenation of BZ, compared to Pd and Pt. In contrast, it outperformed Pt- and Pd-based HPS in production of CHA during AN hydrogenation.

Synthesized Pt-, Pd-, and Ru-containing HPS-based catalysts were shown to be highly active and selective in hydrogenation of both nonsubstituted and substituted arenes. Catalytic properties of synthesized samples are promising in comparison with some other reported results [32]. For example, hydrogenation of BZ and TL proceeded with 100% selectivity to CH and MCH, respectively, while using Pt NPs supported on HPS or HPS-NR_2_. For comparison, in the case of zeolite-supported catalysts, isomerization took place, resulting in the formation of methylcyclopentane as the main product [32]. Hydrogenation of NL to TL over Pt/HPS-NR_2_ proceeded with the selectivity of 88% at 94% of NL conversion, which is comparable with some reported results [32], or, in some cases, even higher [33].

Finally, we would like to underline that hyper-cross-linked polymers with various functionalities and cross-linking degrees should be used as catalytic supports for different metal NPs and should be tested in liquid-phase hydrogenations of arenes, giving much attention to catalyst stability in view of their multiple reuse. This work is in progress and will be reported elsewhere.

## 4. Materials and Methods

### 4.1. Materials

HPS (Purolite Int., Llantrisant, UK), used as catalyst support, was of two types: hyper-cross-linked nonfunctionalized polystyrene, Macronet MN270 (BET SSA 1337 m^2^/g), and HPS containing tertiary amino groups, MN100 (BET SSA 724 m^2^/g), designated here as HPS-NR_2_. Both supports were washed with distilled water and acetone and then dried under vacuum as described elsewhere [34]. Benzene (BZ, 99.8%), toluene (TOL, 99.8%), naphthalene (NL, 99%), aniline (AN, 99%), anthracene (ANC, 97%), phenylacethylene (PHA, 98%), phenol (PL, ≥99%), propiophenone (PP, 98%), diphenylamine (DPA, 99%), tetrahydrofuran (THF, ≥99.9%), methanol (MeOH, 99.8%), acetone (≥99.5%), hydrogen peroxide (H_2_O_2_, 35%), isopropanol (*i*-PrOH, ≥99.5%), butanol (BuOH, ≥99%), hexane (≥99%), and dodecane (≥99%) were obtained from Sigma-Aldrich. Chloroplatinic acid hydrate (H_2_PtCl_6_·6H_2_O, Pt content 38.41%), palladium acetate (Pd(CH_3_COO)_2_, Pd content 47.68%), and ruthenium hydroxychloride (Ru(OH)Cl_3_, Ru content 45.05%) were purchased from JSC “Aurat” (Moscow, Russia). Sodium hydroxide (NaOH) was obtained from Reakhim (Moscow, Russia). Reagent-grade hydrogen of 99.999% purity was received from AGA (Tver, Russia). All chemicals were used as received. Distilled water was purified with an Elsi-Aqua water purification system.

### 4.2. Catalyst Synthesis and Characterization

Pt-, Pd-, and Ru-containing HPS-based catalysts were synthesized via wet-impregnation according to procedure, which is described elsewhere [34,35,36]. In a typical experiment, 1 g of pretreated, dried, and crushed (<63 μm) granules of HPS were impregnated with 2.8 mL of the THF solution of precursor (H_2_PtCl_6_·6H_2_O or Pd(CH_3_COO)_2_) of a chosen concentration. The HPSs impregnated with Pt or Pd were dried, washed with distilled water until reaching a neutral pH, and dried again at 70 °C until the constant weight was achieved. In the case of Ru/HPS, a complex solvent (THF, MeOH, and water in a volumetric ratio of 7:1:1) with dissolved therein calculated amount of Ru(OH)Cl_3_ was used for impregnation. The Ru-containing HPS was dried at 70 °C for 1 h, the dried catalyst was boiled in aqueous solution of NaOH (concentration of 0.1 mol/L) at continuous stirring, and then H_2_O_2_ was added. The resulting catalyst was washed with distilled water until neutral pH was reached, and dried again at 70 °C.

Thus, the catalysts Pt/HPS, Pt/HPS-NR_2_, Pd/HPS-NR_2_, and Ru/HPS-NR_2_ were synthesized containing either 1 wt.% or 2 wt.% of noble metals. Before the experiments, all the catalyst samples were activated in a hydrogen flow (100 mL/min) at 300 °C for 3 h. Such activation has no influence on the HPS structure [11] (see also Figure S1) and results in formation of NPs of metallic Pd or Pt and RuO_2_ (see the data of XPS below) in a micro–mesoporous polymeric environment (BET SSA for all the samples was in the range 700–1000 m^2^/g).

**Figure 14:**
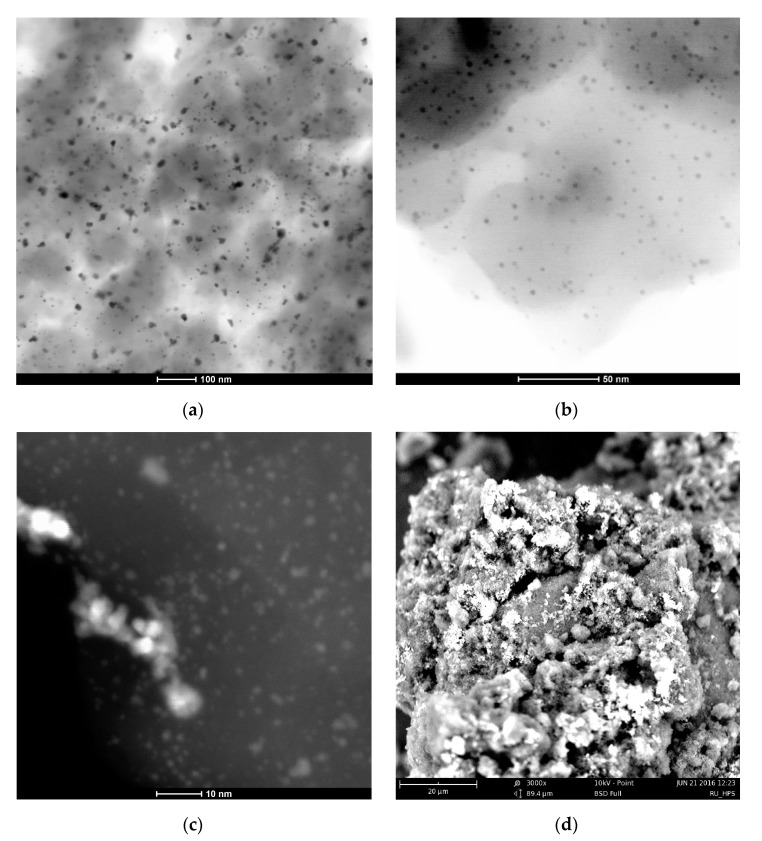
HAADF-STEM images of activated samples ((**a**) 1% Pt/HPS-NR_2_ (scale 100 nm), (**b**) 2% Pd/HPS-NR_2_ (scale 50 nm), (**c**) 2% Ru/HPS-NR_2_ (scale 10 nm)) and SEM image (**d**) of 2% Ru/HPS-NR_2_ (scale 20 μm).

Figure 14 shows STEM data using the example of the 1% Pt/HPS-NR_2_, 2 wt.% Pd/HPS-NR_2_, and 2% Ru/HPS-NR_2_ catalysts. It was found that among these three samples, Pd NPs were the most evenly distributed (Figure 14b) and had mean diameter of 2.6 ± 0.4 nm, which is typical for HPS impregnated with Pd acetate, irrespective of the polymer type, palladium loading, and reduction method [37,38]. Pt NPs had mean diameter of 3.9 ± 1.7 nm, but also a lot of aggregates with diameters up to 11 nm and higher (Figure 14a). The 2 wt.% Ru/HPS-NR_2_ contained small NPs of RuO_2_ of about 2–4 nm in diameter (Figure 14c), which were located nonuniformly, closer to the outer surface of the polymer granule [39]. See also Figure 14d, where light regions correspond to RuO_2_ (the average content of elements calculated via EDX is the following: carbon 34%, oxygen 41%, ruthenium 25%).

Metal state was confirmed by the XPS analysis. It was shown that palladium is present in the form of metallic NPs (BE of Pd 3d_5/2_ is 335.0 ± 0.1 eV for Pd^0^ NPs and 336.0 ± 0.1 eV for small Pd clusters) and PdO (BE is 337.2 ± 0.1 eV [40]). The metallic form Pd^0^ was predominant on the catalysts’ surface (Figure S2c). The existence of oxide can be explained by the fact that, after the activation in H_2_ flow, the catalysts were stored in air. In the case of 1 wt.% Pt/HPS (Figure S2a) and 2 wt.% Pt/HPS-NR_2_ (Figure S2b), the following values of BE (±0.1 eV) of Pt 4f_7/2_ were found: 71.5 eV (Pt^0^) and 72.9 eV (PtO) [40]. For 1% Pt/HPS-NR_2_ [11], BE of Pt 4f_7/2_ was equal to 71.4 ± 0.1 eV, which corresponds to Pt^0^ [40]. In the case of 2 wt.% Ru/HPS-NR_2_ (Figure S2d), ruthenium was in the form of RuO_2_ (BE is 281.2 ± 0.1 eV) and RuO_2_*nH_2_O (BE is 283.0 ± 0.1 eV).

### 4.3. Reaction Procedure and Analysis of Reaction Mixture

Testing of Pt-, Pd-, and Ru-containing HPS-based samples in hydrogenation of different arenes, with the exception of PHA, was carried out in a stainless-steel autoclave reactor (Parr Instruments, Moline, IL, USA) having an internal volume of 80 mL. In a typical experiment, 0.05 g (or 0.1 g) of preliminarily activated catalyst was placed in the reactor. Then, substrate and 40 mL of solvent were added. After that, the reactor was sealed, purged with nitrogen, and heated under stirring (1500 rpm) up to operating temperature. After reaching working temperature, the first sample of the reaction mixture was taken. Then, nitrogen was replaced with hydrogen, overall working pressure was attained (time “zero” for the reaction), and the reaction started. Sampling of the reaction mixture was carried out via long narrow capillary (internal diameter of 0.1 mm); the volume of each sample was 200 μL.

In the case of PHA, hydrogenation was carried out in a 60 mL isothermal glass batch reactor installed in a shaker at vigorous stirring (more than 800 two-sided shakings per minute). The total volume of the liquid phase was 30 mL. A recirculating bath (LOIP LT 100, Saint Petersburg, Russia) was used to stabilize the reaction temperature within ±1 °C, with water as a heating medium. The reactor was connected to a gasometrical burette for online hydrogen consumption control. At the beginning of each experiment, the temperature was stabilized, the reactor was charged with catalyst, and the catalyst was kept under hydrogen for 60 min. Then PHA was added, and reaction was started.

Samples of the reaction mixture were analyzed via GC (Kristallux 4000M) equipped with FID and capillary column ZB-WAX (60 m × 0.53 mm i.d., 1 μm film thickness) or GC–MS (Shimadzu GCMS-QP2010S) equipped with a capillary column HP-1MS (100 m × 0.25 mm i.d., 0.50 μm film thickness). Helium was used as a carrier gas. The concentrations of the reaction mixture components were calculated using absolute calibration method using chemically pure components (in the case of GC) or using the internal standard calibration method (DPA was used as an internal standard) (in the case of GC–MS).

Conversion of substrates was defined as

*X* (%) = (*C_sub_*_,0_ − *C_sub,i_*) × *C_sub_*_,0_^−1^ × 100. (1)

Selectivity with respect to target product was given as

*S* (%) = *C_prod,i_* × (*C_sub_*_,0_ − *C_sub,i_*)^−1^ × 100. (2)

Initial reaction rate was designated as *R*_0_, [mol_sub_∙mol_Me^−1^∙s^−1^_]:

*R*_0_ = (*C_sub_*_,0_ − *C_sub,i_*) × (*C_Me_* × *τ_i_*)^−1^. (3)

### 4.4. Catalyst Characterization Methods

HPS-based catalysts were characterized by liquid nitrogen physisorption, diffuse reflectance infrared Fourier transform spectroscopy (DRIFTS), X-ray photoelectron spectroscopy (XPS), and scanning transmission electron microscopy (STEM).

Liquid nitrogen physisorption was carried out using a Beckman Coulter SA 3100 (Coulter Corporation, Miami, FL, USA). Prior to the analysis, each sample was placed in a quartz cell installed in the Becman Coulter SA-PREP. The samples were pretreated over 60 min under nitrogen at 120 °C. Once the pretreatment was completed, the cell was cooled and weighed, and then transferred to the analytical port. Analysis was performed at −196 °C and at relative pressure of 0.9814 (for pores less than 100 nm in diameter) to obtain a PSD (ADS) profile.

DRIFTS analysis was carried out using an IRPrestige-21 FTIR spectrometer (Shimadzu, Kyoto, Japan) equipped with a DRS-8000 diffuse reflectance accessory (Shimadzu, Japan). The background sample was a mirror of the material of the optical system of the DRS-8000 accessory. All spectra were recorded in the 4000–500 cm^−1^ range of wavenumbers at a resolution of 4 cm^−1^.

XPS data were obtained using Mg Kα (hν = 1253.6 eV) radiation with an ES-2403 spectrometer (Institute for Analytic Instrumentation of RAS, Saint Petersburg, Russia) equipped with an energy analyzer PHOIBOS 100-MCD5 (SPECS, Berlin, Germany) and X-ray source XR-50 (SPECS, Berlin, Germany). All the data were acquired at X-ray power of 250 W. Survey spectra were recorded at an energy step of 0.5 eV with an analyzer pass energy of 40 eV, and high-resolution spectra were recorded at an energy step of 0.05 eV with an analyzer pass energy of 7 eV. Samples were outgassed for 180 min before analysis and were stable during the examination. The data analysis was performed via CasaXPS.

STEM characterization was carried out using an FEI Tecnai Osiris instrument (Thermo Fisher Scientific, Waltham, MA, USA) operating at an accelerating voltage of 200 kV, equipped with a high-angle annular dark field (*HAADF*) detector (Fischione, Export, PA, USA) and an energy-dispersive X-ray (*EDX*) microanalysis spectrometer (EDAX, Mahwah, NJ, USA). Samples were prepared by embedding the catalyst in epoxy resin followed by microtomming (*ca*. 50 nm thick) at ambient temperature. For the image processing, DigitalMicrograph (Gatan, Pleasanton, CA, USA) software and TIA (Thermo Fisher Scientific, Waltham, MA, USA) were used. Holey carbon/Cu grid was used as a sample support.

## Figures and Tables

**Table 1:** Data of catalytic testing of HPS-based catalysts in hydrogenation of nonpolar aromatics (*C_S_*_,0_ = 0.28 mol/L, catalyst loading 100 mg, temperature 230 °C, overall pressure 5 MPa, reaction duration 60 min).

N	Catalyst	Substrate	Product	Solvent	X (S), %	*R*_0_, mol_sub_/(mol_Me_ × s)
1	1% Pt/HPS	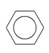 (BZ)	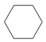 (CH)	hexane	96 (100)	1.37
2	1% Pt/HPS ^(1)^			hexane	98 (100)	0.77
3	1% Pt/HPS 2nd use ^(1)^			hexane	84 (100)	0.43
4	1% Pt/HPS-NR_2_			dodecane	38 (100)	0.47
5	2% Pt/HPS-NR_2_			*i*-PrOH	90 (100)	0.90
6				dodecane	92 (100)	0.89
7				hexane	100 (100)	1.66
8	2% Ru/HPS-NR_2_			hexane	89 (100)	0.40
9	2% Pd/HPS-NR_2_			hexane	100 (100)	0.82
10	1% Pt/HPS-NR_2_^(2)^	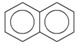 (NL)	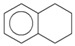 (TL)	hexane [11]	94 (88)	0.15
11	1% Pt/HPS-NR_2_	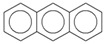 (ANC)	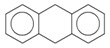 (9,10-DANC)	dodecane	82 (88)	0.85
12	1% Pt/HPS-NR_2_	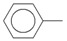 (TOL)	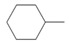 (MCH)	dodecane	80 (100)	1.41

^(1)^*C_BZ_*_,0_ = 0.14 mol/L. ^(2)^ Temperature 240 °C.

**Table 2:** Data of catalytic testing of HPS-based catalysts in hydrogenation of substituted aromatics.

N	Catalyst	Substrate	Product	Solvent	T, °C	P, MPa	X (S), %	*R*_0_, mol_sub_/(mol_Me_ × s)
1	1% Pt/HPS	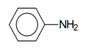 (AN)	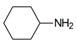 (CHA)	hexane	150	2	100 (55) ^1^	0.32
2	1% Pt/HPS 2nd use			hexane	150	2	100 (56) ^1^	0.21
3	2% Pt/HPS-NR_2_			hexane	150	3	41 (51) ^2^	0.32
4	2% Ru/HPS-NR_2_			hexane	150	3	54 (87) ^2^	0.05
5	2% Pd/HPS-NR_2_			hexane	150	3	58 (60) ^2^	0.07
6	2% Pd/HPS-NR_2_	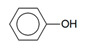 (PL)	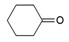 (CHN)	water	150	2	96 (40) ^3^	0.24
7				EtOH	150	2	41 (60) ^3^	0.07
8				BuOH	150	2	53 (64) ^3^	0.12
9	1% Pd/HPS-NR_2_	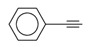 (PHA)	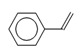 (STY)	TOL	90	ambient	100 (88) ^4^	6.70
10				EtOH	65	ambient	100 (78) ^4^	1.25
11				*i*-PrOH	70	ambient	100 (69) ^4^	2.25
12	1% Pd/HPS-NR_2_	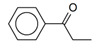 (PP)	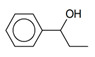 (PPL)	hexane	80	0.25	92 (98) ^5^	0.13

^1^*C_AN_*_,0_ = 0.14 mol/L, catalyst loading 100 mg, values of conversion (X) and selectivity (S) are indicated for the reaction time 120 min. ^2^ *C_AN_*_,0_ = 0.41 mol/L, catalyst loading 100 mg, values of X and S are indicated for the reaction time 150 min. ^3^ *C_PL_*_,0_ = 0.21 mol/L, catalyst loading 50 mg, 2 MPa is partial hydrogen pressure, values of X and S are indicated for the reaction time 90 min. ^4^ *C_PHA_*_,0_ = 0.48 mol/L, catalyst loading 30 mg, maximum values of X and corresponding S are indicated. ^5^ *C_PP_*_,0_ = 0.09 mol/L, values of X and S indicated for the reaction time 150 min.

## Data Availability

Data sharing is not applicable to this article.

## Supplementary Materials

The following are available online, Figure S1: DRIFT spectra of 2% Pt/HPS-NR_2_ (a), 2% Pd/HPS-NR_2_ (b), and 2% Ru/HPS-NR_2_ (c): initial (black line) and after activation in hydrogen flow (red line), Figure S2: High-resolution XPS spectra of Pt 4f (a,b), Pd 3d (c), and Ru 3d (d) in the activated samples: 1% Pt/HPS (a), 2% Pt/HPS-NR_2_ (b), 2% Pd/HPS-NR_2_ (c), and 2%-Ru/HPS-NR_2_ (d).

## Sample Availability

Samples of the HPS-based catalysts are available from the authors.

## Abbreviations

AN, aniline; ANC, anthracene; BZ, benzene; CH, cyclohexane; CHA, cyclohexylamine; CHN, cyclohexanone; 9,10-DANC, 9,10-dihydroanthracene; diphenylamine, DPA; HPS, hyper-cross-linked polystyrene; MCH, methylcyclohexane; NL, naphthalene; NP, nanoparticle; PHA, phenylacethylene; PL, phenol; PP, propiophenone, PPL, phenylpropanol; STY, styrene; TL, tetralin; TOL, toluene.
