# Supplementary material for: Noble Metal Nanoparticles Stabilized by Hyper-Cross-Linked Polystyrene as Effective Catalysts in Hydrogenation of Arenes
Source: Molecules. 2021 Aug 3;26(15):4687. doi: 10.3390/molecules26154687 (PMC8348316; doi:10.3390/molecules26154687)
Supplement: Supplementary file 1 [file molecules-26-04687-s001.zip › molecules-1302000-supplementary.pdf]

Supplementary Materials

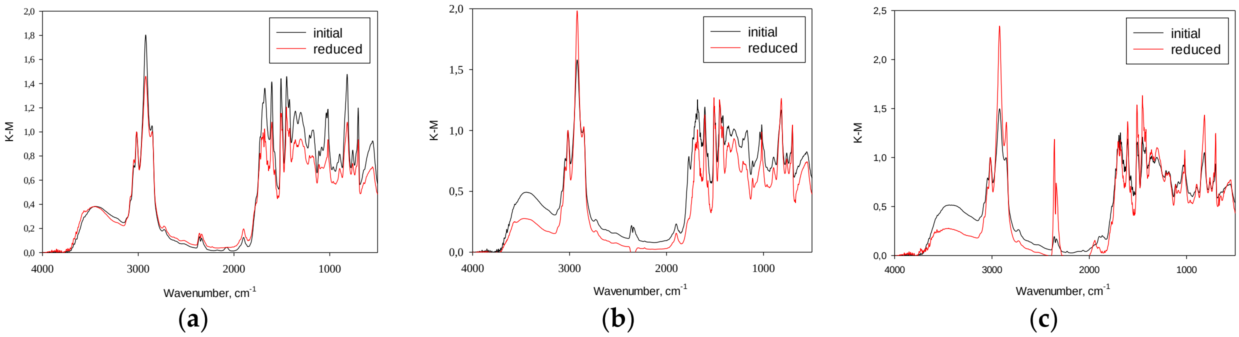

Figure S1. DRIFT spectra of 2%-Pt/HPS-NR_2 (a), 2%-Pd/HPS-NR_2 (b) and 2%-Ru/HPS-NR_2 (c): initial (black line) and after activation in hydrogen flow (red line).

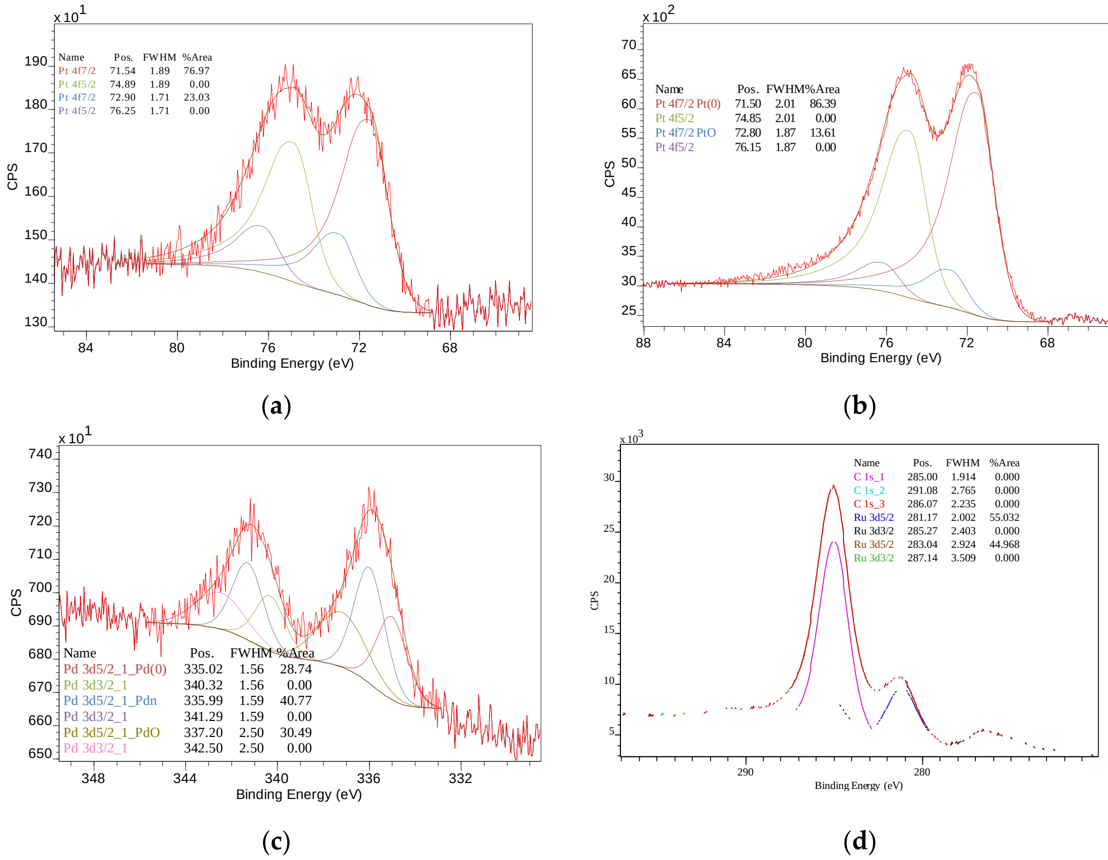

Figure S2. High-resolution XPS spectra of Pt 4f (a, b), Pd 3d (c) and Ru 3d (d) in the activated samples: 1%-Pt/HPS (a), 2%-Pt/HPS-NR_2 (b), 2%-Pd/HPS-NR_2 (c) and 2%-Ru/HPS-NR_2 (d).
